# Indole-3-Propionic Acid Disrupts Glycolytic Adaptation and Promotes Oxidative Stress-Associated Injury in Oral Squamous Cell Carcinoma

**DOI:** 10.3390/ijms27198867

**Published:** 2026-10-05

**Authors:** Negar Taghavi Pourianazar

**Affiliations:** Medical Laboratory Techniques, Vocational School of Health Services, Istanbul Aydin University, 34295 Istanbul, Turkey; negarpourianazar@aydin.edu.tr or negartaghavi22@gmail.com

**Keywords:** oral squamous cell carcinoma, indole-3-propionic acid, microbiota-derived metabolite, metabolic plasticity, reactive oxygen species

## Abstract

Oral squamous cell carcinoma (OSCC) shows aggressive invasion, frequent recurrence, and marked metabolic adaptability. Microbial tryptophan metabolites are increasingly recognized as potential modulators of cancer-related signaling; however, the direct role of indole-3-propionic acid (IPA) in OSCC remains poorly defined. This study examined whether IPA suppresses malignant fitness in HSC-3 and SCC-25 cells while using OKF6/TERT-1, an immortalized human oral keratinocyte cell line, as a non-malignant comparator where biologically appropriate. HSC-3 and SCC-25 cells were treated with vehicle or IPA. Based on the calculated IC50 values from 72 h viability assays, fixed concentrations of 300 µM IPA for HSC-3 cells and 400 µM IPA for SCC-25 cells were selected for subsequent functional experiments. OKF6/TERT-1 cells were used as a non-malignant comparator for baseline transcriptional analyses. Metabolic activity was assessed by the MTS assay. Cytotoxicity and cell death-related stress were additionally examined using lactate dehydrogenase release and colorimetric caspase-3 activity assays. Migration was determined by a wound-healing assay. Metabolic adaptation was evaluated by glucose consumption, lactate secretion, conditioned-medium pH, intracellular adenosine triphosphate, spectrophotometric intracellular reactive oxygen species measurement, and quantitative real-time polymerase chain reaction. Baseline profiling showed that glycolysis-associated transcripts were consistently higher in HSC-3 and SCC-25 cells than in OKF6/TERT-1 cells, with the strongest malignant shift observed in HSC-3. IPA (300 µM for HSC-3, 400 µM for SCC-25) reduced 72 h viability to 55.3% in HSC-3 and 64.8% in SCC-25 cells. IPA increased intracellular reactive oxygen species by 1.86-fold in HSC-3 and 1.49-fold in SCC-25 cells, increased caspase-3 activity, and elevated lactate dehydrogenase release. In parallel, glucose consumption, lactate secretion, adenosine triphosphate content, and migration decreased, whereas conditioned-medium pH and CDH1 expression increased. IPA may directly suppress OSCC cell survival, motility, and glycolytic adaptation while promoting oxidative stress-associated injury and modulating epithelial–mesenchymal transition markers. This metabolic framework may provide a novel experimental direction for OSCC research.

## 1. Introduction

Oral squamous cell carcinoma (OSCC) is the dominant histologic subtype of oral cancer and one of the most prevalent malignancies worldwide, accounting for more than 300,000 new cases annually [1,2]. Despite decades of research and improvements in multimodal treatment strategies, including surgery, radiation, and platinum-based chemotherapy, the five-year survival rate for OSCC remains approximately 50–60%, a figure that has changed little over the past three decades [1,2]. This persistently poor prognosis reflects the biological complexity of OSCC, including its local aggressiveness, propensity for regional lymph node dissemination, high recurrence rates, and the significant functional morbidity associated with treatment [3]. Biologic heterogeneity and adaptive tumor behavior continue to limit durable disease control, underscoring the urgent need to identify novel therapeutic targets and mechanistically distinct intervention strategies.

One of the hallmarks supporting OSCC progression is metabolic plasticity, particularly the ability to maintain glycolytic flux, lactate export, and survival under microenvironmental stress [4,5]. This metabolic reprogramming, broadly characterized by the Warburg effect, enables cancer cells to sustain rapid proliferation and biosynthetic activity while evading immune surveillance and therapeutic interventions. In OSCC, overexpression of glucose transporter 1 (GLUT1) and key glycolytic enzymes, including hexokinase 2 (HK2), lactate dehydrogenase A (LDHA), and monocarboxylate transporter 4 (MCT4), has been consistently associated with advanced tumor stage, lymph node metastasis, and poor overall survival [4,5,6]. Notably, Kunkel et al. demonstrated that GLUT1 expression correlates with tumor aggressiveness and is an independent prognostic factor in OSCC patients, highlighting the clinical relevance of glycolytic targeting [6]. The resulting acidic tumor microenvironment not only sustains metabolic plasticity but also facilitates epithelial–mesenchymal transition (EMT), a transcriptional reprogramming process that endows cancer cells with enhanced migratory and invasive capabilities [5,7].

EMT in OSCC is orchestrated by a network of transcription factors, including SNAIL, SLUG, and ZEB1, which collectively suppress epithelial markers such as E-cadherin (CDH1) and upregulate mesenchymal proteins, including vimentin and N-cadherin [7,8]. Loss of CDH1 expression is a well-established driver of OSCC invasiveness; studies by Pyo et al. and Lo et al. have demonstrated that CDH1 downregulation correlates with lymph node metastasis, recurrence, and reduced disease-free survival in oral cancer cohorts [8,9]. Importantly, emerging evidence suggests that metabolic reprogramming and EMT are not independent processes but are mechanistically intertwined: lactate-driven extracellular acidification activates SNAIL-mediated CDH1 suppression, while HIF-1α, stabilized under glycolytic conditions, directly transcribes EMT-promoting genes [5,7]. This metabolic–EMT axis represents a compelling yet underexplored therapeutic target in OSCC.

This adaptive phenotype is increasingly being interpreted within the context of host–microbe interactions. Recent literature has highlighted the oral microbiota and its metabolites as active participants in epithelial transformation, inflammatory remodeling, and tumor progression in OSCC [10,11]. The oral cavity harbors a complex ecosystem of bacterial, fungal, and viral species that collectively influence local immune homeostasis, epithelial barrier function, and carcinogenic processes. Dysbiosis of the oral microbiome may involve alterations in bacterial, fungal, and viral communities. In OSCC, enrichment of bacterial taxa such as *Fusobacterium nucleatum*, *Porphyromonas gingivalis*, and *Treponema denticola* has been associated with disease development and progression, while fungal components, including *Candida* spp., have also been discussed in relation to dysbiotic oral environments and carcinogen-related processes [10,11,12,13]. However, most studies have focused on broad microbial associations or on a limited set of metabolites, whereas the direct experimental interrogation of less-studied microbial metabolites in OSCC remains relatively sparse [10,11,12,13,14]. This gap represents a significant opportunity to identify novel therapeutic agents derived from the microbiota–host interface.

Among these candidates, indole metabolites generated through microbial tryptophan catabolism are emerging as biologically relevant regulators of epithelial homeostasis, oxidative stress, and cancer-associated signaling [14,15]. Indole derivatives, including indole-3-propionic acid (IPA), indole-3-aldehyde (I3A), and indole-3-carbinol (I3C), have demonstrated diverse biological activities across multiple cancer models. In particular, IPA has received increasing attention because of its well-documented antioxidant and aryl hydrocarbon receptor (AhR)-mediated signaling properties [14,15,16]. IPA is a microbial tryptophan metabolite whose production has been primarily associated with intestinal bacteria, including the gut-resident species *Clostridium sporogenes* [10,11,12]. IPA has been shown to exert AhR-mediated signaling effects in epithelial cells—a pathway linked to cancer cell metabolism and EMT. Antitumor effects of IPA have been reported in prostate, colorectal, and hepatocellular carcinoma models; for instance, Huang et al. demonstrated that IPA suppresses prostate cancer cell proliferation through apoptosis concurrent with the activation of the p53 signaling pathway, while Bersier et al. reported that IPA inhibits colorectal cancer cell migration [15,16]. IPA has been shown to function through the pregnane X receptor (PXR) and AhR, inducing gene expression profiles that modulate oxidative stress responses and inflammatory signaling in cancer cells [17]. In breast cancer models, IPA demonstrated cytostatic properties by activating AhR and PXR receptors, reducing cancer stem cell proportions, and inducing oxidative and nitrosative stress through the downregulation of NRF2 and upregulation of inducible nitric oxide synthase (iNOS) [18]. These diverse mechanistic findings across multiple cancer types suggest that IPA operates through interconnected pathways encompassing oxidative stress modulation, cell cycle regulation, and metabolic disruption. Notably, the PI3K/AKT and AMPK/mTOR signaling cascades represent key downstream targets potentially regulated by IPA-mediated metabolic perturbation, although their direct involvement in IPA-induced antitumor effects in OSCC remains to be experimentally confirmed.

This knowledge gap is particularly relevant because OSCC progression depends not only on proliferation but also on the ability to preserve clonogenic survival, withstand oxidative and metabolic stress, sustain motile behavior, and acquire invasive capacity through EMT. To address this gap, the present study evaluated whether IPA suppresses OSCC malignant fitness in HSC-3 and SCC-25 cells—two OSCC cell lines representing biologically distinct phenotypes—while incorporating OKF6/TERT-1 cells as a non-malignant reference to assess the selectivity of IPA-induced cytotoxicity through a coordinated disruption of metabolic adaptation and modulation of epithelial–mesenchymal transition (EMT) markers. The expanded in vitro panel included MTS viability testing, lactate dehydrogenase (LDH) release, caspase-3 activity, intracellular reactive oxygen species (ROS) measurement, migration assay, biochemical metabolic assays, and quantitative real-time PCR to provide comprehensive mechanistic insight into IPA-mediated suppression of OSCC cell fitness. IPA was evaluated as an exogenously applied compound in OSCC cell cultures.

## 2. Results

### 2.1. Baseline Profiling Identified a More Glycolysis-Prone and Motile Phenotype in HSC-3 Cells

**Figure 1:**
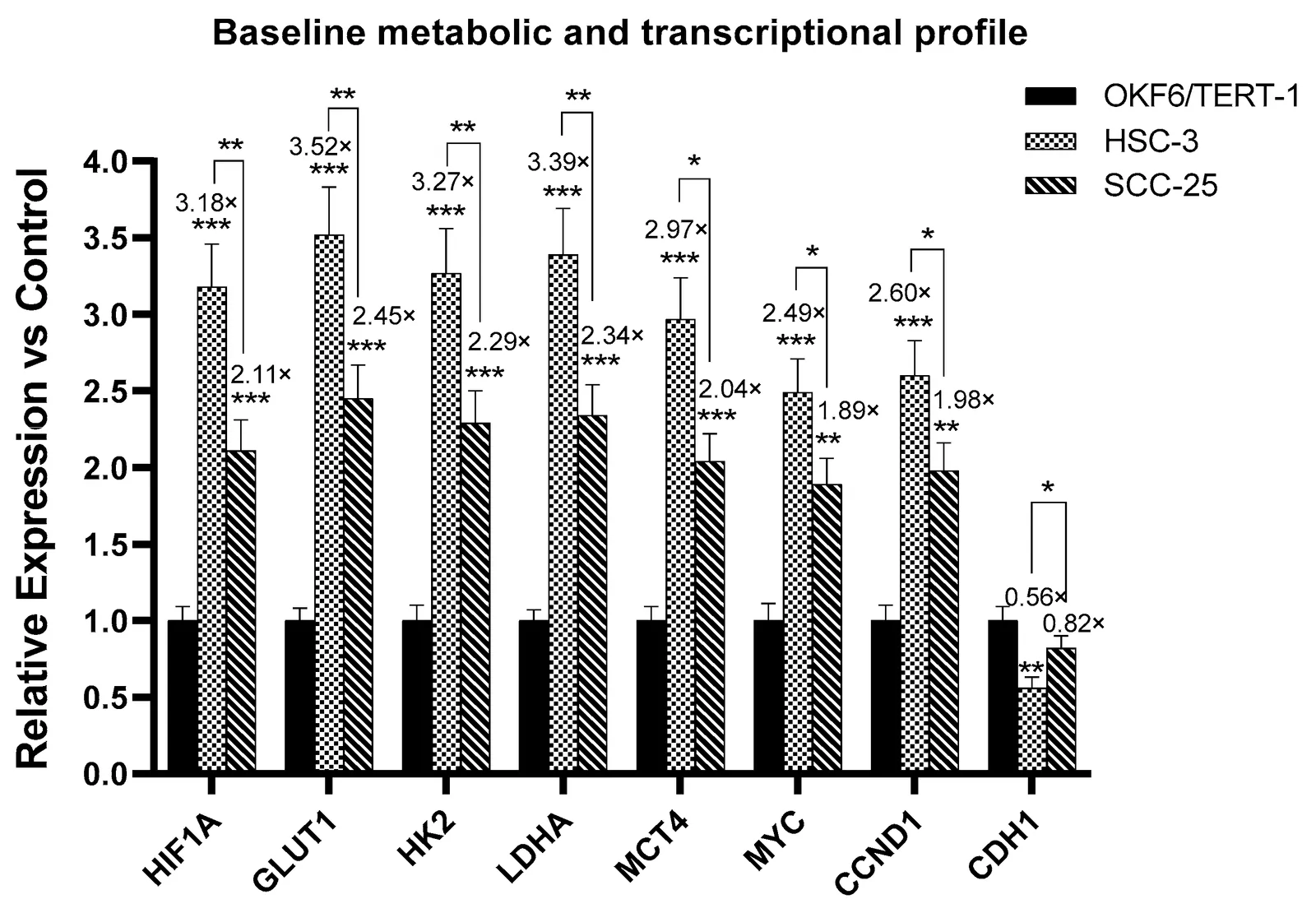
Baseline metabolic and transcriptional profile of HSC-3 and SCC-25 cells. The figure shows the baseline relative expression of HIF1A, GLUT1, HK2, LDHA, MCT4, MYC, CCND1, and CDH1 in OKF6/TERT-1, HSC-3, and SCC-25 cells. Glycolysis-associated genes (HIF1A, GLUT1, HK2, LDHA, MCT4) and proliferation-related genes (MYC, CCND1) are markedly upregulated in both OSCC cell lines relative to OKF6/TERT-1 cells, with HSC-3 cells displaying a more aggressive metabolic phenotype. In contrast, CDH1 (E-cadherin) expression is suppressed in both cell lines, with more pronounced suppression in HSC-3 cells, reflecting a more advanced epithelial–mesenchymal transition state. Data are presented as mean ± SD (n = 3), and all treatment groups in each cell line were compared with the control group. Statistical significance: * *p* < 0.05, ** *p* < 0.01, *** *p* < 0.001 compared to control.

Baseline quantitative real-time polymerase chain reaction analysis demonstrated that both OSCC cell lines exhibited a transcriptional pattern consistent with malignant metabolic reprogramming when compared with OKF6/TERT-1 cells (Figure 1). Relative to the non-malignant comparator, HSC-3 cells showed marked upregulation of HIF1A (3.18 ± 0.28-fold), GLUT1 (3.52 ± 0.31-fold), HK2 (3.27 ± 0.29-fold), LDHA (3.39 ± 0.30-fold), MCT4 (2.97 ± 0.27-fold), MYC (2.49 ± 0.22-fold), and CCND1 (2.60 ± 0.23-fold), whereas CDH1 expression declined to 0.56 ± 0.07-fold. SCC-25 cells displayed the same directional pattern but with consistently lower amplitude, including HIF1A at 2.11 ± 0.20-fold, GLUT1 at 2.45 ± 0.22-fold, HK2 at 2.29 ± 0.21-fold, LDHA at 2.34 ± 0.20-fold, MCT4 at 2.04 ± 0.18-fold, MYC at 1.89 ± 0.17-fold, and CCND1 at 1.98 ± 0.18-fold, while CDH1 remained comparatively less suppressed at 0.82 ± 0.08-fold. Taken together, these data indicate that both cell models retain a glycolysis-oriented OSCC phenotype, but HSC-3 cells occupy a more aggressive biological state characterized by stronger glycolytic programming and weaker epithelial restraint.

### 2.2. IPA Decreased MTS-Defined Metabolic Activity in OSCC Cells

**Figure 2:**
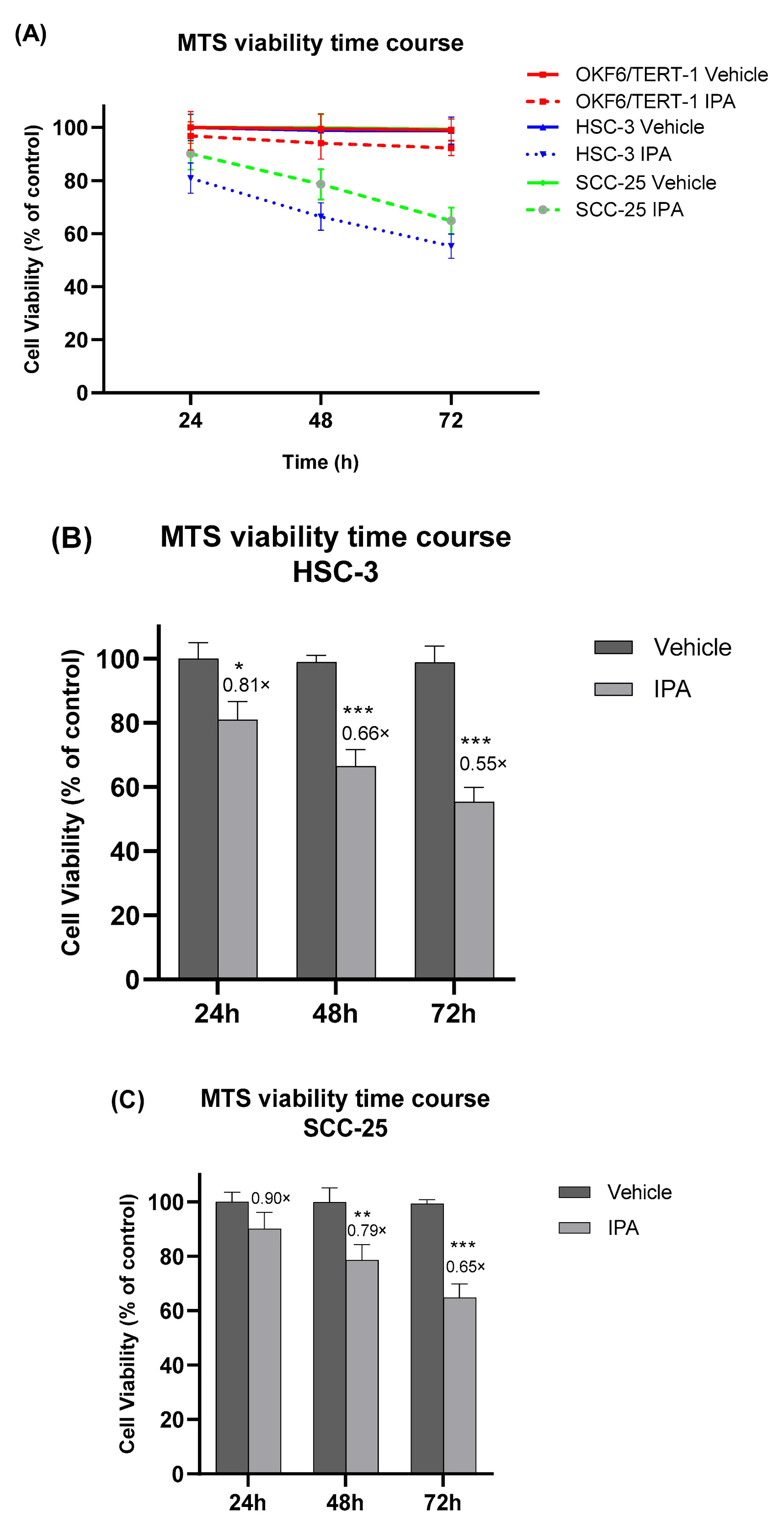

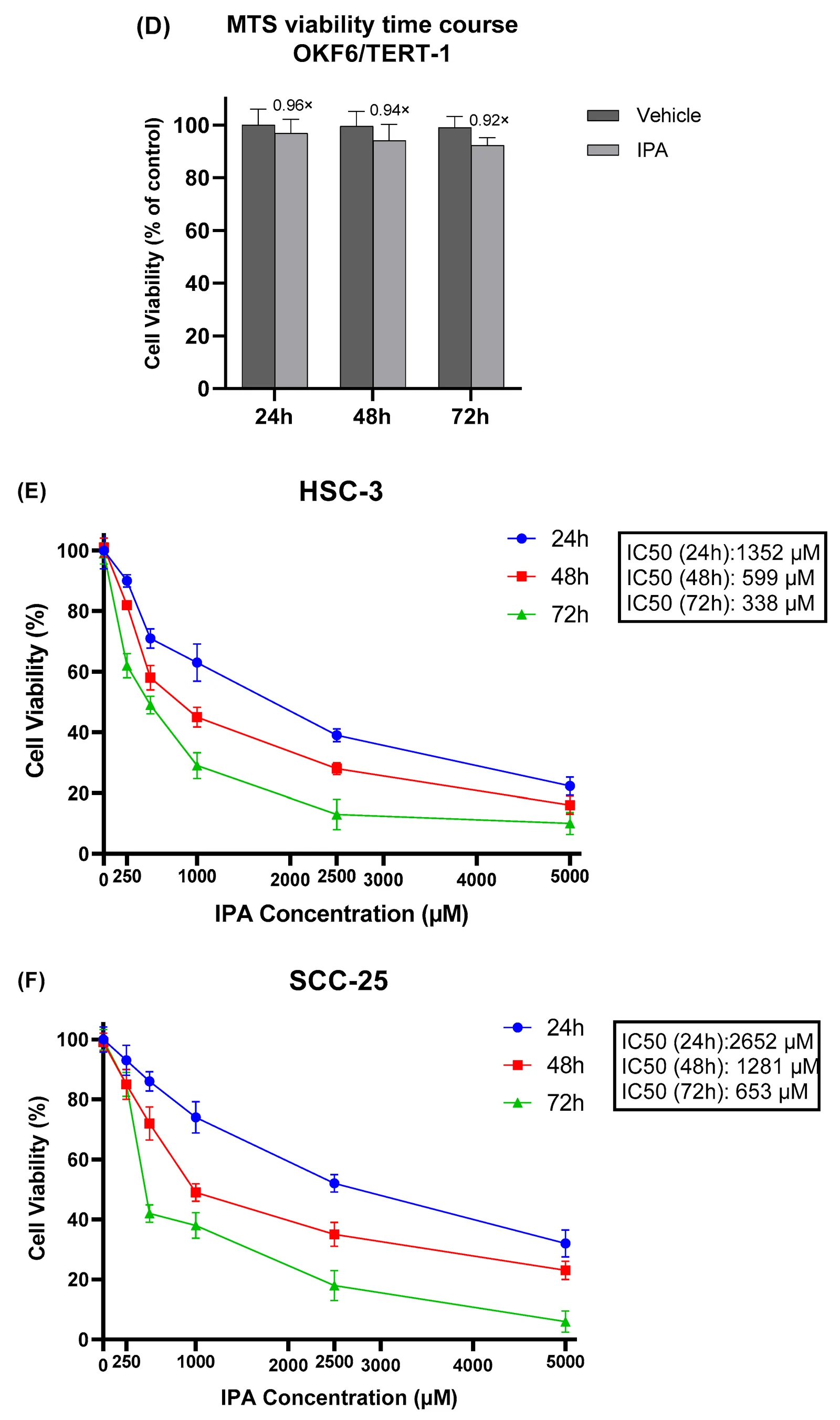

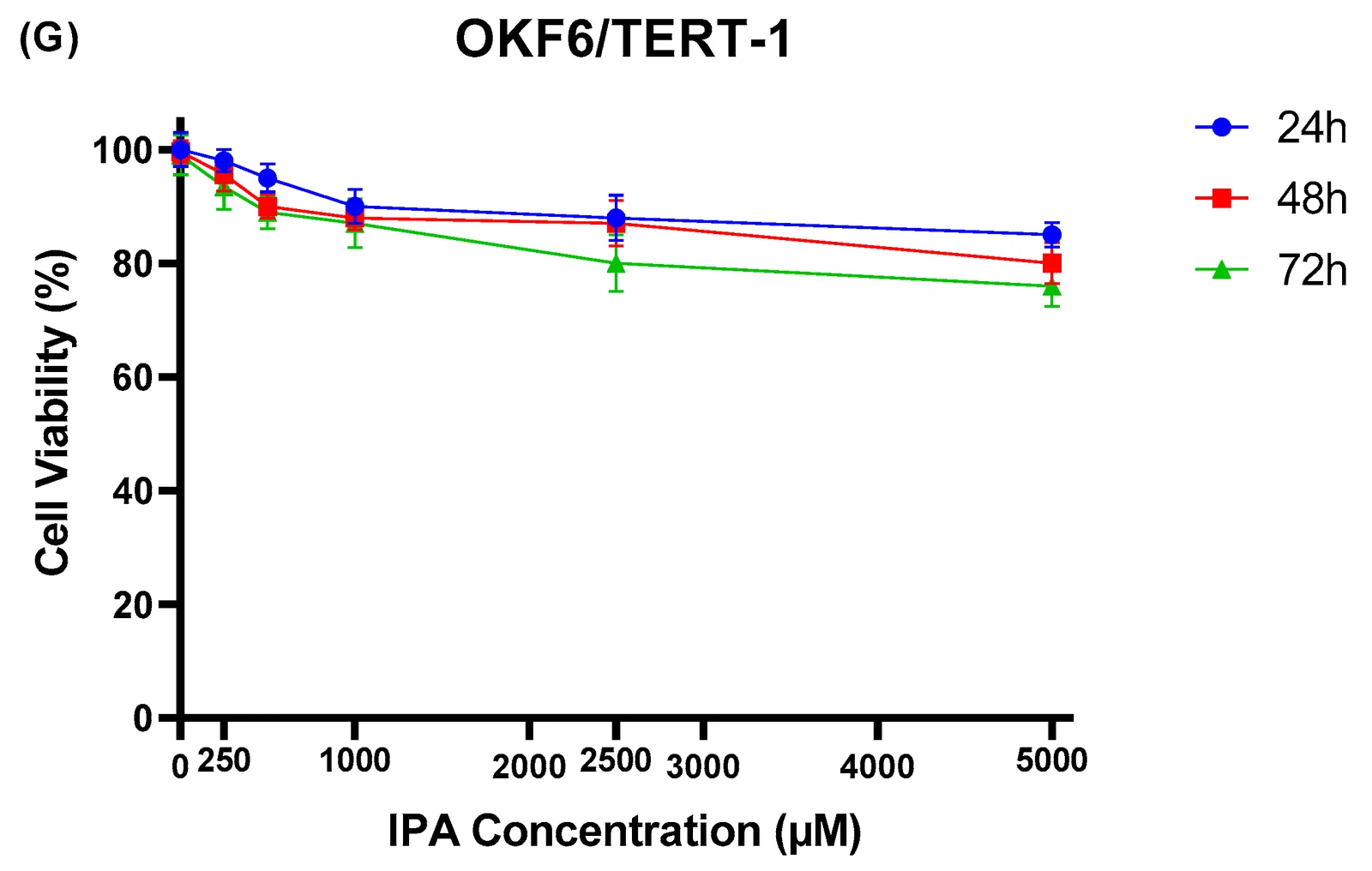
Concentration- and time-dependent effects of IPA on MTS-defined metabolic activity. HSC-3, SCC-25, and OKF6/TERT-1 cells were exposed to indole-3-propionic acid (IPA) for 24, 48, or 72 h. (**A**) Comparative time-course analysis of MTS-defined metabolic activity in HSC-3, SCC-25, and OKF6/TERT-1 cells treated with vehicle or the corresponding IPA concentrations. (**B**) MTS-defined viability of HSC-3 cells at 24, 48, and 72 h following exposure to 300 µM IPA. Viability values relative to vehicle control were 0.81 ± 0.06 at 24 h, 0.66 ± 0.05 at 48 h, and 0.55 ± 0.05 at 72 h. (**C**) MTS-defined viability of SCC-25 cells at 24, 48, and 72 h following exposure to 400 µM IPA. Viability values relative to vehicle control were 0.90 ± 0.06 at 24 h, 0.79 ± 0.06 at 48 h, and 0.65 ± 0.05 at 72 h. (**D**) MTS-defined viability of OKF6/TERT-1 cells at 24, 48, and 72 h following IPA exposure, showing only a limited reduction in MTS-defined metabolic activity under the tested conditions. (**E**) HSC-3, (**F**) SCC-25, and (**G**) OKF6/TERT-1 cells were treated with increasing concentrations of IPA (0–5000 µM) for 24 h (blue), 48 h (red), or 72 h (green), and MTS-defined metabolic activity was measured. Data points represent the mean ± SD of three independent biological experiments. IC_50_ values were calculated by nonlinear regression using a four-parameter logistic model with a variable slope. The calculated IC_50_ values for HSC-3 cells were 1352 µM at 24 h, 599 µM at 48 h, and 338 µM at 72 h. The corresponding IC_50_ values for SCC-25 cells were 2652 µM, 1281 µM, and 653 µM, respectively. For OKF6/TERT-1 cells, the IC_50_ was not reached within the tested concentration range and was therefore estimated to be >5000 µM at all timepoints. These findings indicate a differential MTS-defined response to IPA between the OSCC cell lines and OKF6/TERT-1 cells under the tested conditions; they do not establish cancer selectivity for the downstream functional or metabolic endpoints. Data are presented as mean ± SD of three independent biological replicates. Statistical significance was determined relative to the corresponding vehicle-treated control; * *p* < 0.05, ** *p* < 0.01, and *** *p* < 0.001.

Exposure to IPA (300 µM for HSC-3 and 400 µM for SCC-25) reduced metabolic viability in both OSCC cell lines in a time-dependent manner (Figure 2). In HSC-3 cells, MTS-defined viability decreased from 100.0 ± 3.2% at baseline to 80.9 ± 5.7% at 24 h, 66.4 ± 5.2% at 48 h, and 55.3 ± 4.6% at 72 h, corresponding to progressive reductions of 19.1%, 33.6%, and 44.7% relative to vehicle. SCC-25 cells were also responsive, although the magnitude of suppression was smaller, with viability values of 90.1 ± 6.0%, 78.6 ± 5.7%, and 64.8 ± 5.0% at 24, 48, and 72 h, respectively, representing reductions of 9.9%, 21.4%, and 35.2% compared to vehicle control. Importantly, OKF6/TERT-1 cells exposed to identical IPA concentrations demonstrated minimal viability loss, with values of 96.3 ± 3.2% at 24 h, 94.1 ± 3.8% at 48 h, and 92.3 ± 4.2% at 72 h, representing only 3.7%, 5.9%, and 7.7% reductions, respectively. This pattern indicates that IPA exerts a sustained and selective inhibitory effect on metabolic activity in transformed OSCC cells, with a more pronounced impact in the intrinsically more aggressive HSC-3 model, while sparing immortalized human oral keratinocyte cells. The corresponding dose–response curves and IC50 calculations for all cell lines at each timepoint are provided in Figure 2.

### 2.3. IPA Increased Oxidative Stress, Membrane Injury, and Caspase-3-Associated Response

**Figure 3:**
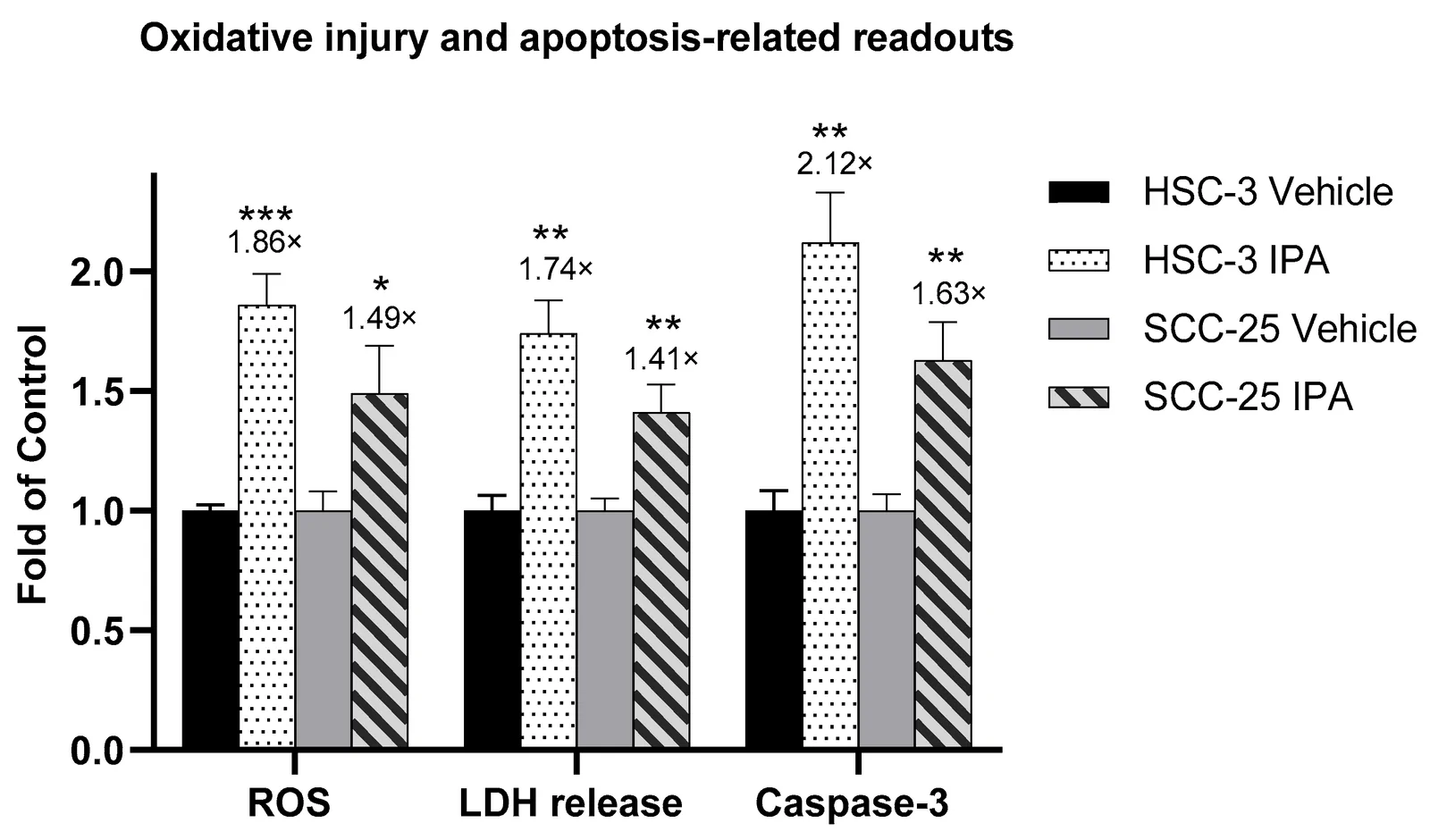
IPA promotes oxidative stress-associated injury. The graph presents intracellular ROS, LDH release, and caspase-3 activity. Data are presented as mean ± SD (n = 3), and all treatment groups in each cell line were compared with the vehicle group. Statistical significance: * *p* < 0.05, ** *p* < 0.01, *** *p* < 0.001 compared to control.

To clarify whether treatment-associated growth suppression was accompanied by cellular injury, we examined intracellular ROS production, LDH release, and caspase-3 activity (Figure 3). IPA increased ROS accumulation to 1.86 ± 0.15-fold of control in HSC-3 cells and to 1.49 ± 0.13-fold in SCC-25 cells, indicating a consistent increase in oxidative burden in both backgrounds. In parallel, LDH release rose to 1.74 ± 0.14-fold in HSC-3 cells and 1.41 ± 0.12-fold in SCC-25 cells, supporting enhanced membrane damage and cytotoxic stress. Caspase-3 activity showed the strongest treatment-associated shift, increasing to 2.12 ± 0.18-fold in HSC-3 cells and to 1.63 ± 0.14-fold in SCC-25 cells. The combined pattern of ROS accumulation, LDH release, and caspase-3 activation suggests that IPA exposure does not merely slow proliferation but also drives oxidative stress-associated injury with a caspase-3-associated response execution component. Notably, the stronger response observed in HSC-3 cells across all three endpoints is consistent with the notion that the more metabolically active and motile phenotype is also more vulnerable to IPA-induced stress (Figure 3).

### 2.4. IPA Altered Bulk Metabolic Readouts Under the Tested Conditions

**Figure 4:**
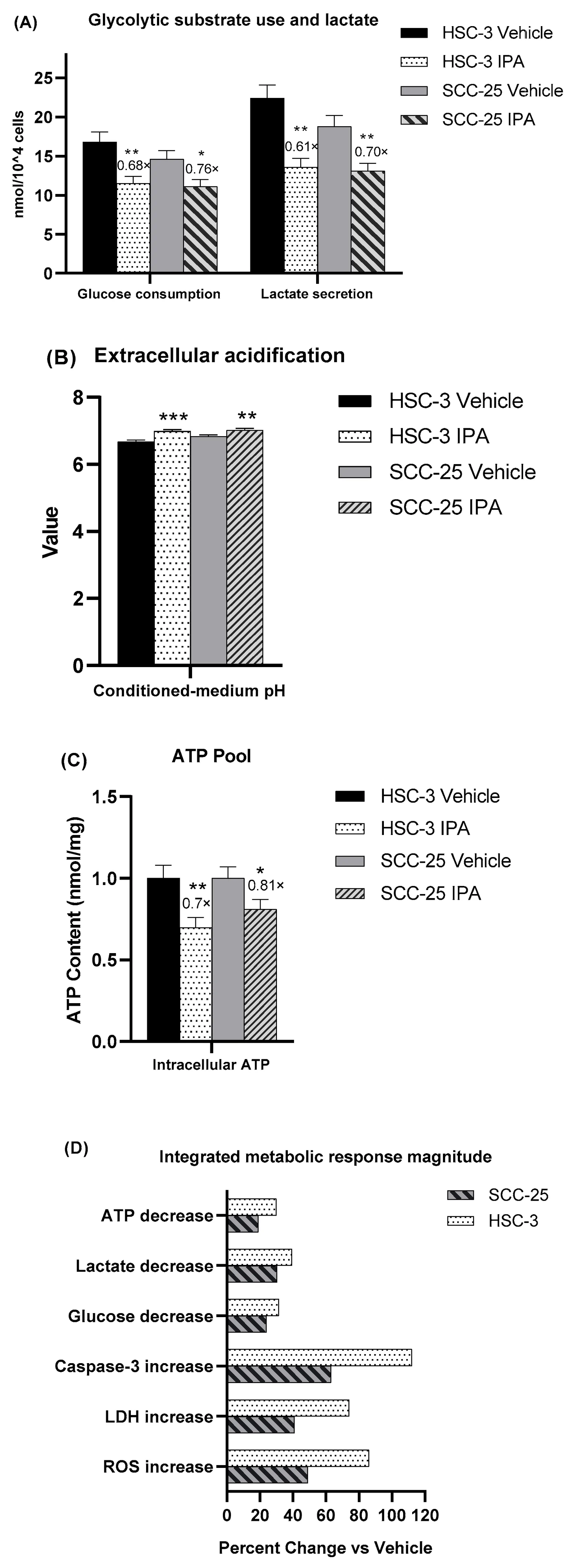
IPA-associated changes in bulk metabolic readouts. (**A**) Glucose consumption and lactate secretion in HSC-3 and SCC-25 cells treated with vehicle (control) or fixed concentrations of IPA (300 µM for HSC-3 and 400 µM for SCC-25). (**B**) Conditioned-medium pH and (**C**) intracellular ATP content after IPA treatment. (**D**) Summary of the relative magnitude of oxidative and metabolic responses in the two cell lines, showing HSC-3 cells display greater sensitivity to IPA-induced metabolic disruption. Statistical significance between HSC-3 and SCC-25 responses was evaluated using Student’s t-test. These measurements were obtained under fixed initial seeding conditions and were not normalized to DNA or total protein measured at harvest; therefore, they should be interpreted as bulk biochemical readouts rather than definitive per-cell measurements of glycolytic flux. Conditioned-medium pH is presented as a bulk-medium parameter and not as a direct measurement of extracellular proton flux. Data are presented as mean ± SD of three independent biological replicates, and all treatment groups in each cell line were compared with the vehicle group. Statistical significance: * *p* < 0.05, ** *p* < 0.01, *** *p* < 0.001 compared to control.

Because OSCC progression is closely linked to glycolytic plasticity, I next examined whether IPA (300 µM for HSC-3 and 400 µM for SCC-25) altered key biochemical indicators of energy metabolism (Figure 4A–C). In HSC-3 cells, glucose consumption declined from 16.8 ± 1.3 to 11.5 ± 0.9 nmol per 10^4^ cells, whereas lactate secretion decreased from 22.4 ± 1.7 to 13.6 ± 1.1 nmol per 10^4^ cells. In SCC-25 cells, glucose consumption fell from 14.6 ± 1.1 to 11.1 ± 0.9 nmol per 10^4^ cells and lactate secretion from 18.8 ± 1.4 to 13.1 ± 1.0 nmol per 10^4^ cells. These metabolic reductions were accompanied by a clear shift in conditioned-medium pH toward less acidic values, increasing from 6.68 ± 0.04 to 6.99 ± 0.04 in HSC-3 cells and from 6.83 ± 0.05 to 7.02 ± 0.05 in SCC-25 cells. Intracellular ATP content also declined, reaching 0.70 ± 0.06 relative units in HSC-3 cells and 0.81 ± 0.06 relative units in SCC-25 cells after treatment. Because treatment-related differences in viable cell number may influence bulk biochemical measurements, glucose consumption, lactate secretion, conditioned-medium pH, and intracellular ATP were interpreted cautiously. IPA treatment was associated with reduced glucose consumption, reduced lactate secretion, and lower intracellular ATP content in HSC-3 and SCC-25 cells, as summarized in Figure 4D. Conditioned-medium pH shifted toward less acidic values under the tested conditions. However, because these measurements were not normalized to DNA or total protein at harvest, the findings do not establish a per-cell reduction in glycolytic flux. Moreover, conditioned-medium pH was not interpreted as a direct measurement of extracellular proton flux or acidification rate.

### 2.5. IPA Repressed Glycolysis-Associated Transcripts, Increased CDH1 Expression, and Suppressed Migratory Activity

**Figure 5:**
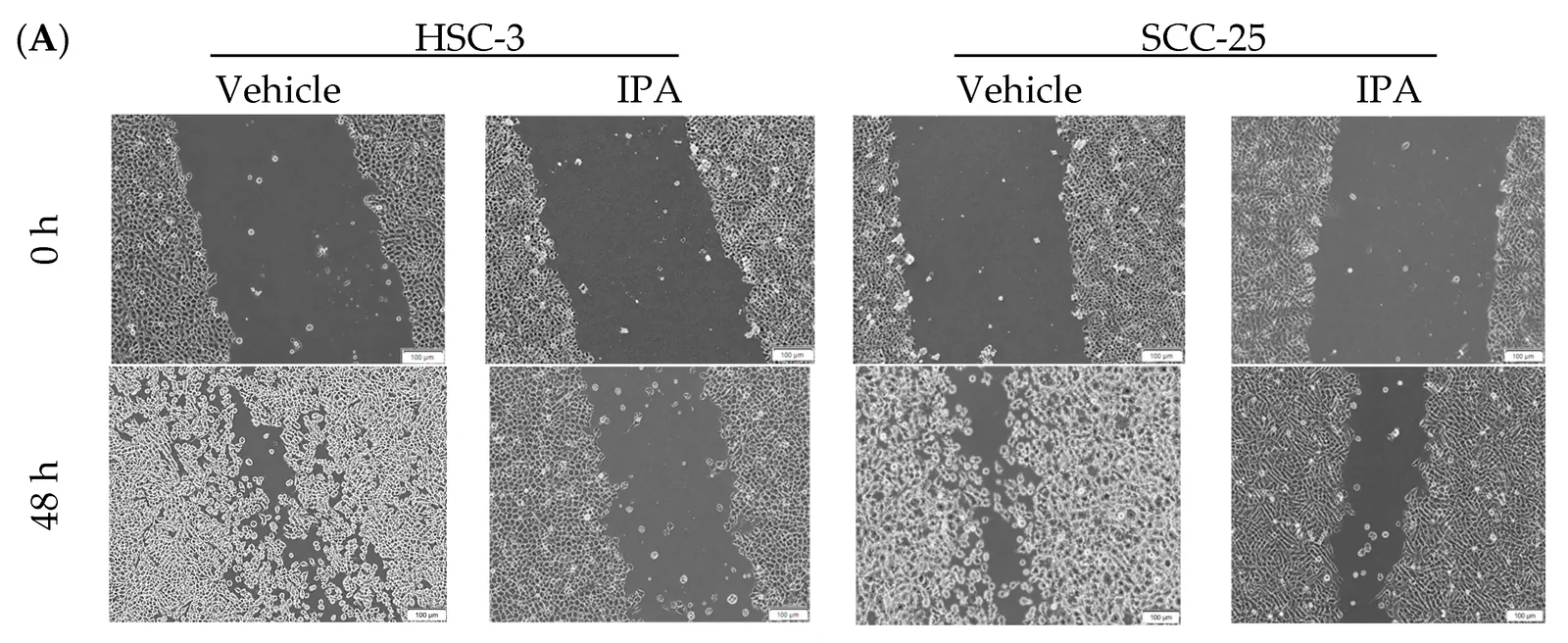

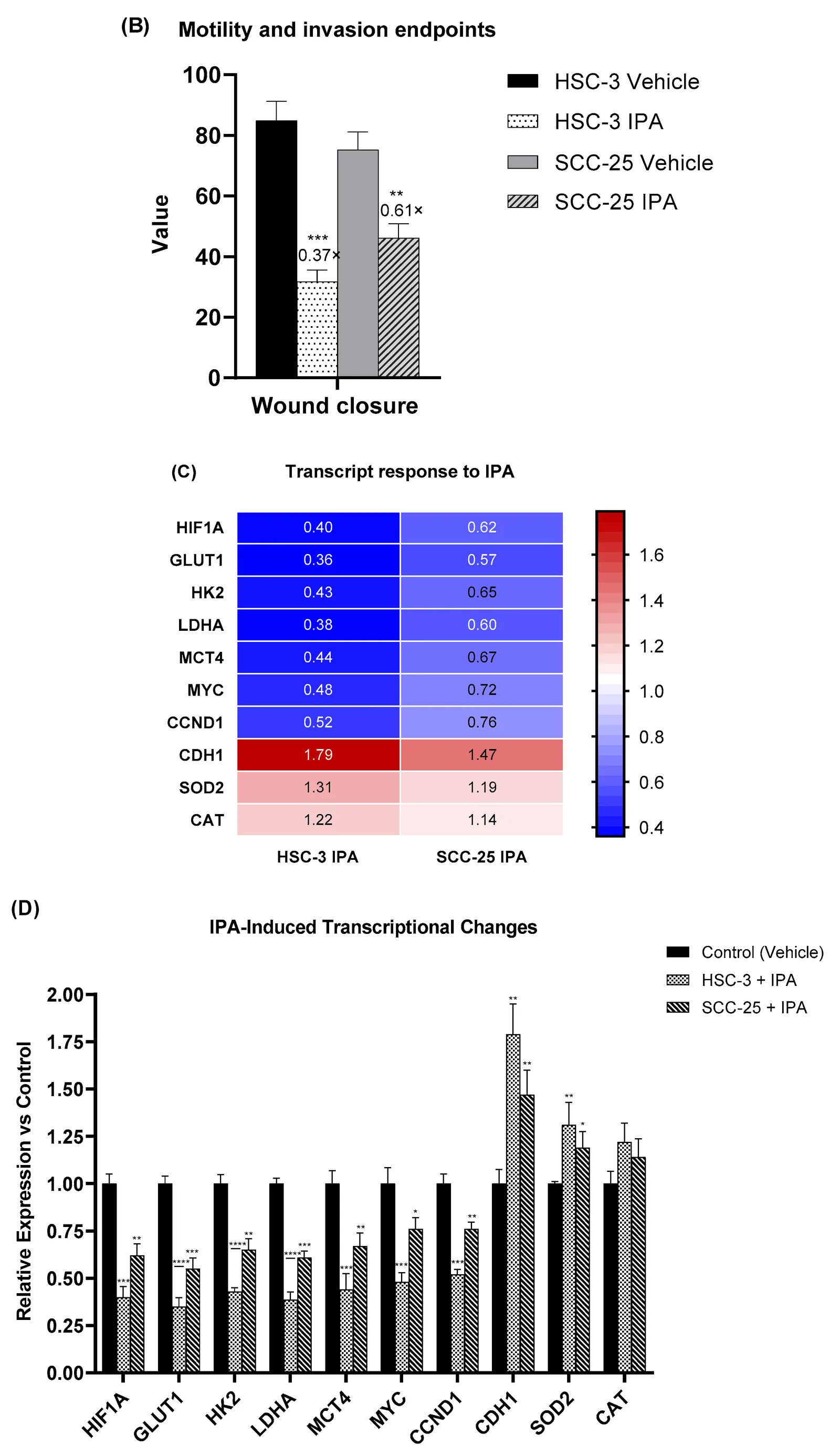

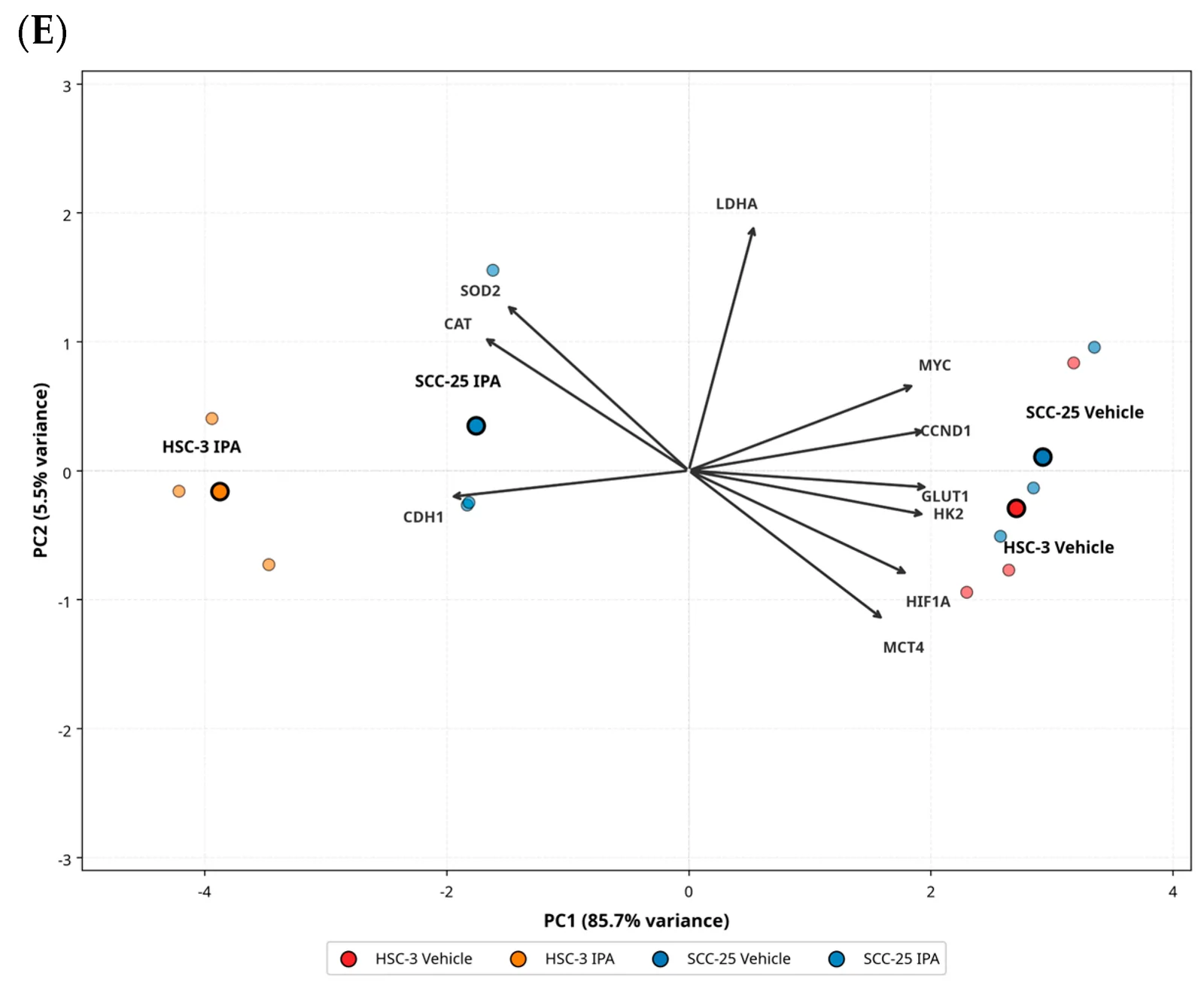
IPA represses motility and reshapes the transcriptional program of OSCC cells. (**A**) Representative phase-contrast microscopic images of a wound-healing assay in HSC-3 and SCC-25 cells at 0 h and 48 h following treatment with vehicle control or IPA (300 µM for HSC-3 and 400 µM for SCC-25). A uniform scratch was introduced into confluent monolayers, and wound closure was monitored over 48 h. In HSC-3 cells, wound closure at 48 h decreased from 84.9 ± 6.3% (vehicle) to 31.8 ± 3.8% (IPA), whereas in SCC-25 cells, wound closure decreased from 75.2 ± 5.9% (vehicle) to 46.1 ± 4.7% (IPA). Scale bar = 100 µm. Data are representative of three independent experiments performed in triplicate. (**B**) Wound-healing assay showing migration capacity of HSC-3 and SCC-25 cells treated with vehicle (control) or IPA at 0 h and 48 h. (**C**) Quantitative real-time PCR analysis showing transcript-level changes in glycolysis-associated genes (HIF1A, GLUT1, HK2, LDHA, MCT4), stress-response genes (SOD2, CAT), and a motility-related gene (CDH1) after IPA treatment. (**D**) Summary of IPA-induced transcriptional changes across functional gene categories, demonstrating coordinated suppression of glycolytic and pro-motility genes with simultaneous restoration of epithelial markers. (**E**) Principal component analysis (PCA) biplot of the qPCR gene expression dataset. PC1 (85.7% variance) separates vehicle and IPA-treated groups; PC2 (5.5% variance) reflects gene-specific response patterns. Glycolysis-associated gene loadings (HIF1A, GLUT1, HK2, LDHA, MCT4, MYC, CCND1) are oriented toward vehicle controls, while stress-response gene loadings (CDH1, SOD2, CAT) are oriented toward IPA-treated samples. Data are presented as mean ± SD (n = 3), and all treatment groups in each cell line were compared with the vehicle group. Statistical significance: * *p* < 0.05, ** *p* < 0.01, *** *p* < 0.001, **** *p* < 0.0001 compared to control.

Transcript-level analyses after treatment reinforced the biochemical findings (Figure 5). In HSC-3 cells, IPA reduced HIF1A to 0.40 ± 0.05-fold, GLUT1 to 0.36 ± 0.04-fold, HK2 to 0.43 ± 0.05-fold, LDHA to 0.38 ± 0.04-fold, MCT4 to 0.44 ± 0.05-fold, MYC to 0.48 ± 0.05-fold, and CCND1 to 0.52 ± 0.05-fold of control. In contrast, CDH1 increased to 1.79 ± 0.16-fold, while SOD2 and CAT showed modest upregulation to 1.31 ± 0.12-fold and 1.22 ± 0.11-fold, respectively, consistent with a compensatory antioxidant response. SCC-25 cells displayed a similar but attenuated pattern, with HIF1A, GLUT1, HK2, LDHA, MCT4, MYC, and CCND1 decreasing to 0.62 ± 0.06-fold, 0.57 ± 0.06-fold, 0.65 ± 0.06-fold, 0.60 ± 0.06-fold, 0.67 ± 0.07-fold, 0.72 ± 0.07-fold, and 0.76 ± 0.07-fold, while CDH1 increased to 1.47 ± 0.13-fold and SOD2/CAT rose to 1.19 ± 0.11-fold and 1.14 ± 0.10-fold. Functional motility assay paralleled these transcriptional shifts. Wound closure at 48 h decreased from 84.9 ± 6.3% to 31.8 ± 3.8% in HSC-3 cells and from 75.2 ± 5.9% to 46.1 ± 4.7% in SCC-25 cells. Together, these findings support a coordinated response in which IPA suppresses migratory behavior while simultaneously shifting the transcriptional program away from glycolytic aggressiveness and toward a less motile phenotype. Principal component analysis of the complete transcriptional dataset further confirmed this coordinated shift, demonstrating clear separation between vehicle and IPA-treated groups along the first principal component (PC1; 85.7% of total variance), with glycolysis-associated gene loadings oriented toward vehicle controls and stress-response gene loadings oriented toward IPA-treated samples (Figure 5E).

## 3. Discussion

The present study provides novel experimental evidence that exogenously applied IPA, a microbial tryptophan metabolite primarily associated with intestinal bacterial production, suppresses the malignant fitness of OSCC cells through the coordinated disruption of metabolic adaptation and modulation of EMT markers. Although oral microbiome dysbiosis has been increasingly associated with OSCC pathogenesis, direct mechanistic investigations of specific indole metabolites in OSCC remain limited [10,11,12]. By incorporating OKF6/TERT-1 cells alongside the aggressive HSC-3 and moderate SCC-25 OSCC cell lines, this study demonstrated that IPA exerts a selective anti-cancer effect. IPA progressively reduced MTS-defined viability in both OSCC cell lines, with the intrinsically more aggressive HSC-3 model exhibiting greater sensitivity. The selection of HSC-3 and SCC-25 cell lines was guided by their representation of biologically distinct OSCC phenotypes. HSC-3 is characterized by a highly aggressive, glycolysis-dependent, and motile profile, making it a stringent model for evaluating IPA efficacy under conditions of maximal metabolic stress. SCC-25, in contrast, exhibits a moderate proliferative and glycolytic phenotype, providing a comparative framework to assess the breadth of IPA’s suppressive capacity. By employing both cell lines, this study demonstrates that IPA is effective not only against moderately aggressive OSCC cells but also against the more resilient HSC-3 model, which is often associated with poor clinical outcomes. This comparative approach strengthens the translational relevance of the findings, suggesting that IPA-mediated metabolic disruption may benefit a broad spectrum of OSCC patients. Importantly, OKF6/TERT-1 cells exposed to identical IPA concentrations and timepoints maintained near-complete viability, indicating that IPA-induced cytotoxicity is specific to transformed cells rather than representing universal cellular toxicity. This selective vulnerability aligns with emerging literature suggesting that microbial indole derivatives can target cancer-specific metabolic dependencies while sparing normal oral keratinocytes [14,15]. The corresponding dose–response curves and IC50 calculations for all cell lines at each timepoint are provided in Supplementary Figure S1.

The anti-cancer potential of IPA has been increasingly validated across diverse tumor models. Sari et al. demonstrated that IPA exerts cytostatic effects in breast cancer cells by activating AhR and PXR receptors, reducing cancer stem cell proportions, and inducing oxidative and nitrosative stress through the downregulation of NRF2 and upregulation of iNOS [17]. Their findings parallel the oxidative stress-mediated cytotoxicity observed in the present study, suggesting a conserved mechanism across cancer types. Similarly, Huang et al. reported that IPA suppresses prostate cancer cell proliferation, migration, and invasion through activation of the p53-p21-RB signaling axis, leading to cell cycle arrest and apoptosis [15]. While the current study did not directly examine p53 signaling, the robust caspase-3 activation observed in IPA-treated OSCC cells is consistent with the caspase-3-associated mechanisms described in previous IPA studies. The signaling pathways potentially mediating IPA’s effects warrant further consideration. The PI3K/AKT pathway is a central survival signaling cascade in cancer cells, and its dysregulation is frequently associated with metabolic reprogramming and therapy resistance [19]. IPA-mediated disruption of glycolytic flux and ATP depletion may indirectly suppress PI3K/AKT signaling, as the pathway is sensitive to metabolic perturbations [20]. Furthermore, the AMPK/mTOR axis represents a critical metabolic checkpoint in cancer cells. AMPK, acting as an energy sensor, is typically activated under conditions of metabolic stress and ATP depletion, and its activation subsequently inhibits mTOR-mediated protein synthesis and cell growth [21]. The observed reduction in intracellular ATP following IPA treatment suggests that AMPK activation may represent a plausible downstream mechanism, potentially contributing to the suppression of metabolic activity and proliferation. Additionally, mTOR inhibition has been associated with reduced GLUT1 expression and diminished glycolytic activity [22], which aligns with the transcriptional suppression of GLUT1 and glycolysis-associated genes observed in this study. Together, these considerations suggest that IPA may operate through a network of interconnected signaling pathways—including PI3K/AKT, AMPK/mTOR, and AhR/PXR—to disrupt the metabolic and survival machinery of OSCC cells.

A central finding of this study is the paradoxical role of IPA in modulating oxidative stress. Although IPA is widely documented in the literature as a potent antioxidant and free radical scavenger in normal physiological contexts and neuroprotection [16], the results obtained in this study revealed that IPA significantly elevated intracellular ROS in OSCC cells. This ROS accumulation was accompanied by increased LDH release and robust activation of caspase-3, culminating in apoptosis-associated signaling. This apparent paradox can be explained by the context-dependent bioactivity of certain antioxidants in the tumor microenvironment. In malignant cells operating under high metabolic stress and altered redox balance, compounds with antioxidant properties can act as pro-oxidants when their concentrations exceed the buffering capacity of the cell, overwhelming the endogenous antioxidant defenses [23,24]. Similar dual behavior has been observed with other established antioxidants, such as high-dose vitamin C and resveratrol, which selectively induce ROS-mediated apoptosis in cancer cells while protecting normal tissues [25,26]. In this model, the modest compensatory upregulation of SOD2 and CAT transcripts was insufficient to neutralize the IPA-induced ROS surge in OSCC cells. Furthermore, the absence of ROS elevation and cytotoxicity in OKF6/TERT-1 cells reinforces the concept that IPA exploits the unique redox vulnerability of cancer cells to trigger selective oxidative injury.

Beyond oxidative stress, the data strongly support a model in which IPA attenuates glycolytic plasticity, a hallmark of OSCC progression [4,5]. Baseline transcriptional profiling confirmed that HSC-3 and SCC-25 cells heavily rely on glycolysis, exhibiting marked upregulation of HIF1A, GLUT1, HK2, LDHA, and MCT4 compared to normal keratinocytes. Treatment with IPA systematically repressed these glycolysis-associated transcripts, leading to significant functional consequences: reduced glucose consumption, decreased lactate secretion, and diminished intracellular ATP availability. The concurrent shift in conditioned-medium pH toward a less acidic range provides functional validation of reduced glycolytic throughput. The suppression of GLUT1 is particularly noteworthy, as GLUT1 overexpression is a recognized driver of therapy resistance and poor prognosis in OSCC [6,27]. Recent studies have demonstrated that targeted inhibition of GLUT1 not only starves tumor cells of glucose but also enhances their sensitivity to oxidative stress and apoptotic stimuli [28,29]. By simultaneously disrupting glucose influx (via GLUT1) and lactate efflux (via MCT4), IPA effectively collapses the metabolic machinery required for OSCC survival under stress.

The baseline upregulation of GLUT1 and suppression of CDH1 represent two interconnected adaptive mechanisms in OSCC. GLUT1-mediated glucose uptake sustains aerobic glycolysis, providing both ATP and biosynthetic intermediates necessary for rapid proliferation and survival under stress. Simultaneously, CDH1 suppression destabilizes epithelial adherence junctions, facilitating EMT and enhanced motility. Notably, the acidic microenvironment created by elevated lactate secretion further promotes EMT and invasive behavior. The observation in this study that IPA simultaneously represses GLUT1 and restores CDH1 expression while suppressing migration indicates a dual mechanism: IPA disrupts both the glycolytic machinery supporting metabolic plasticity and the transcriptional program driving EMT. This coordinated suppression of metabolic reprogramming and EMT-associated traits suggests a bidirectional feedback mechanism wherein metabolic normalization may facilitate partial restoration of epithelial characteristics [22].

**Figure 6:**
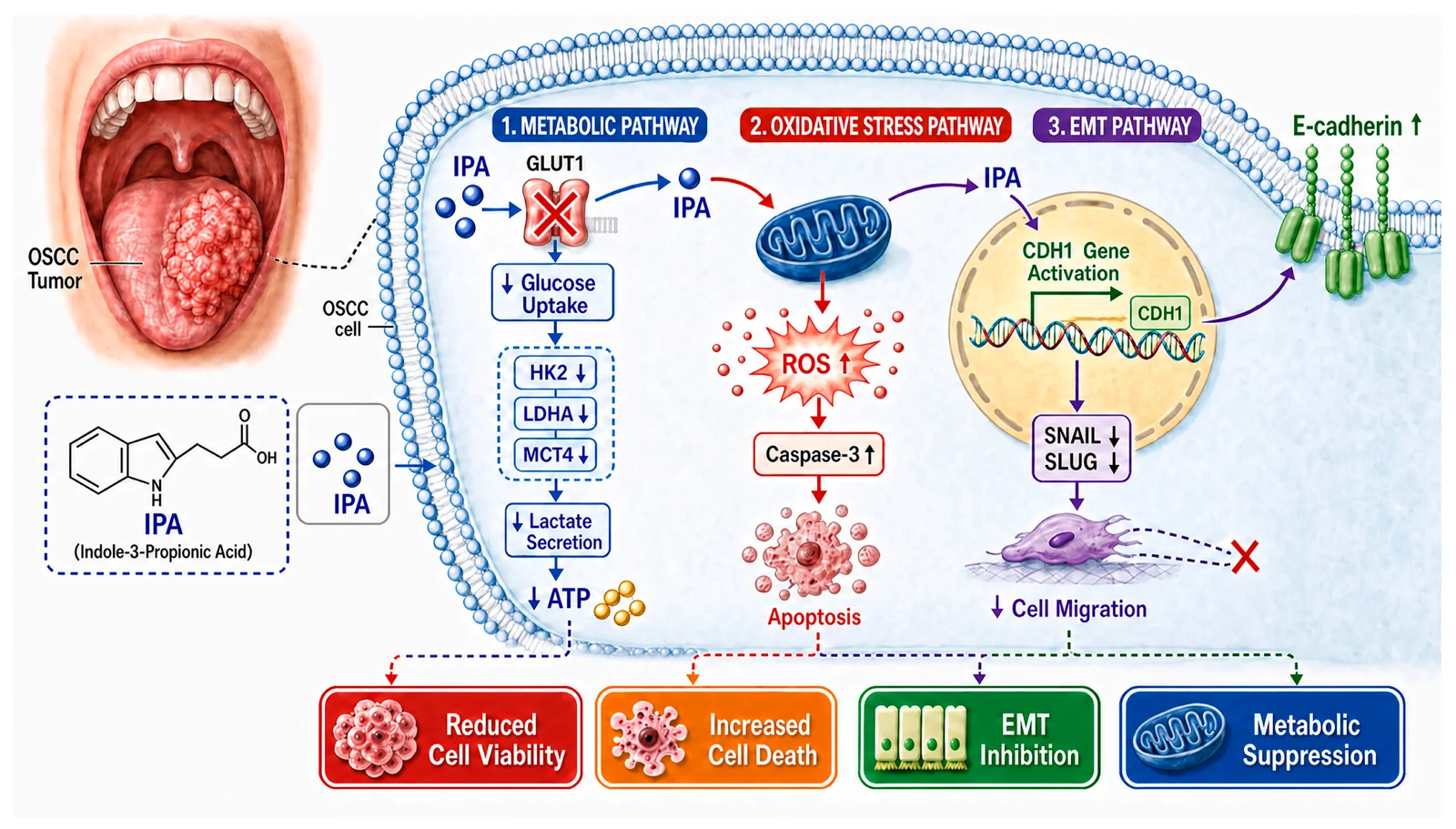
Proposed cellular mechanism of IPA action in OSCC cells. Exposure of OSCC cells to exogenously added IPA, a tryptophan-derived microbial metabolite, is proposed to exert antitumor effects through three interconnected pathways. (1) Metabolic Pathway: IPA inhibits GLUT1-mediated glucose uptake, leading to the downregulation of key glycolytic enzymes (HK2, LDHA, MCT4), reduced lactate secretion, and depletion of intracellular ATP, thereby collapsing the glycolytic machinery that sustains OSCC survival. (2) Oxidative Stress Pathway: IPA induces mitochondrial dysfunction and intracellular ROS accumulation, which triggers caspase-3 activation and cell death. (3) EMT Pathway: IPA activates CDH1 gene expression in the nucleus, partially restoring E-cadherin at the cell membrane, ultimately inhibiting cell migration. The convergence of these three pathways results in reduced cell viability, increased cell death, modulation of EMT markers, and metabolic suppression in OSCC cells. IPA chemical structure (indole ring with propionic acid side chain) is shown in the lower left panel.

The metabolic disruption induced by IPA is intimately linked to the suppression of migratory behavior and the modulation of EMT markers. Baseline analyses revealed pronounced suppression of CDH1 (E-cadherin) in OSCC cells, consistent with their invasive phenotype [8,9]. Remarkably, IPA treatment restored CDH1 expression, leading to a substantial reduction in wound-healing capacity. The acidic tumor microenvironment, primarily driven by excessive lactate secretion, is a known catalyst for EMT and tumor invasion [7,30]. Therefore, the IPA-mediated reduction in lactate production and subsequent attenuation of extracellular acidification likely contributes to the observed EMT reversal. As summarized in the proposed cellular mechanism model (Figure 6), IPA orchestrates a multi-level assault on OSCC cells: it impairs glycolytic energy production, triggers mitochondrial dysfunction and ROS-mediated apoptosis-associated signaling, and restores epithelial adhesion by upregulating E-cadherin. This coordinated suppression of metabolic reprogramming and EMT-associated traits suggests a bidirectional feedback mechanism wherein metabolic normalization facilitates epithelial differentiation [31].

The heightened sensitivity of HSC-3 cells to IPA treatment, as evidenced by greater reductions in viability, more pronounced ROS accumulation, and stronger caspase-3 activation relative to SCC-25 cells, warrants mechanistic interpretation. Baseline transcriptional profiling revealed that HSC-3 cells exhibit a significantly stronger glycolytic dependency, with markedly higher expression of HIF1A, GLUT1, HK2, LDHA, and MCT4 compared to SCC-25 cells. This indicates that HSC-3 cells rely more heavily on aerobic glycolysis for energy production and survival. Furthermore, HSC-3 cells displayed a more suppressed epithelial phenotype, as reflected by lower baseline CDH1 expression, consistent with a more advanced EMT state. Because IPA primarily targets glycolytic machinery—suppressing GLUT1-mediated glucose uptake, downregulating key glycolytic enzymes, and reducing lactate output—the sudden collapse of this primary energy pathway exerts a proportionally greater impact on cells that are more glycolytically addicted. In addition, the higher baseline glycolytic flux in HSC-3 cells generates greater reactive oxygen species as a byproduct of increased metabolic activity, rendering these cells more susceptible to further oxidative stress imposed by IPA. The combined effect of metabolic collapse and amplified oxidative injury explains why HSC-3 cells, despite their aggressive phenotype, are paradoxically more vulnerable to IPA-induced stress than the moderately glycolytic SCC-25 model. This observation is consistent with the concept that cancer cells with the highest metabolic plasticity and glycolytic dependency may represent the most responsive targets for metabolic intervention strategies.

While this in vitro investigation provides evidence that IPA alters OSCC cellular responses, several limitations should be acknowledged. As an in vitro study, the experimental design cannot fully recapitulate the three-dimensional tissue architecture, complex stromal interactions, or immune-cell infiltration of the tumor microenvironment. In addition, in vivo pharmacokinetic, bioavailability, and tissue-distribution data are required to assess the translational relevance of IPA.

The mechanistic conclusions drawn from the transcriptional and biochemical assays also require further validation at the protein level and in functional perturbation models. I did not directly evaluate protein-level changes in the PI3K/AKT, AMPK/mTOR, or p53 signaling pathways by Western blotting. Although the transcriptional and biochemical findings are consistent with alterations in metabolic and survival-related signaling, direct protein-level validation remains an important area for future investigation. Comparative studies using siRNA-mediated knockdown or pharmacological inhibitors targeting PI3K/AKT and AMPK/mTOR signaling would further clarify whether these pathways are required for the IPA-associated response in OSCC cells.

The metabolic and cell-death measurements represent additional limitations. MTS assays measure cellular metabolic activity rather than direct cell death, and therefore reduced MTS-defined metabolic activity may reflect metabolic suppression, reduced cell number, or both. Although increased caspase-3 activity and LDH release were observed after IPA treatment, these findings indicate a caspase-3-associated and membrane-injury-associated response but do not definitively distinguish apoptosis from necrosis. Future studies should therefore include non-metabolic viability and orthogonal cell-death assays, such as Annexin V/PI staining or cleaved PARP analysis.

Glucose consumption, lactate secretion, intracellular ATP content, and conditioned-medium pH were measured under fixed initial seeding conditions but were not normalized to DNA or total protein measured at harvest. Consequently, treatment-related differences in viable cell number may have contributed to the observed bulk biochemical changes, and the present data do not establish a definitive per-cell reduction in glycolytic flux. Conditioned-medium pH was measured under 5% CO_2_ culture conditions and was influenced by the complete culture medium and the presence of IPA; therefore, it was interpreted only as a bulk-medium parameter and not as a direct measure of extracellular proton flux or acidification rate. Future studies should incorporate harvest-time DNA or protein normalization and independent validation using Seahorse extracellular-flux analysis and/or ^13C-tracing.

Moreover, LDH release was not normalized to total cellular LDH, and ROS measurements were normalized to the vehicle control rather than to a cell-independent parameter such as DNA content. These factors should be addressed in future experiments.

The inclusion of OKF6/TERT-1 cells as a non-malignant oral keratinocyte comparator provides useful context for the differential MTS-defined metabolic responses observed between the tested cell models; however, it does not by itself establish cancer-selective cytotoxicity. Finally, future studies using three-dimensional organoid cultures and patient-derived xenograft models will be important to validate these findings and clarify the therapeutic relevance of microbiota-derived indole metabolites in oral oncology.

## 4. Materials and Methods

### 4.1. Cell Lines and Culture Conditions

Human OSCC cell lines HSC-3 and SCC-25 were used to represent biologically distinct oral squamous carcinoma phenotypes. OKF6/TERT-1 immortalized human oral keratinocytes were used as the non-malignant oral epithelial comparator. OKF6/TERT-1 cells were treated in parallel with OSCC cell lines using identical IPA concentrations and timepoints to assess the selectivity of IPA’s anti-cancer activity and to determine whether IPA-induced cytotoxicity was specific to transformed cells or represented universal cellular toxicity. All cell lines used in this study were kindly provided by Professor Dr. Omer Faruk Bayrak of the Department of Medical Genetics, Faculty of Medicine, Yeditepe University. All lines were routinely screened for mycoplasma contamination prior to experimental use. To ensure reproducibility and minimize passage-related phenotypic drift, cells were maintained and utilized exclusively between passages 5 and 20 throughout all experiments. Cells were cultured in Dulbecco’s modified Eagle medium/Ham’s F-12 or supplier-recommended medium supplemented with 10% fetal bovine serum and 1% penicillin-streptomycin at 37 °C in a humidified atmosphere containing 5% carbon dioxide. Cell line authentication and routine mycoplasma testing were performed before experimentation.

### 4.2. IPA Treatment Conditions

IPA (Cat. No. V22550, InvivoChem, North Aurora, IL, USA) was first dissolved in dimethyl sulfoxide (DMSO, MP Biomedicals, Ohio, USA) to prepare a concentrated stock solution (500 mM). This stock solution was then diluted into complete culture medium immediately before use to achieve the desired working concentrations. Complete medium containing serum proteins and other solubilizing components supports a substantially higher effective solubility of IPA than aqueous or low-protein mixtures at equivalent final DMSO content. Range-finding experiments were performed using concentrations from 0 to 5 mM to establish dose–response curves. Based on the calculated IC50 values from the 72 h MTS viability assays, fixed concentrations of 300 µM IPA for HSC-3 cells and 400 µM for SCC-25 cells were selected for subsequent functional experiments to evaluate mechanistic responses without inducing immediate overwhelming cytotoxicity. For the fixed-concentration experiments, 300 µM IPA for HSC-3 cells and 400 µM IPA for SCC-25 cells corresponded to final DMSO concentrations of 0.06% and 0.08% (*v*/*v*), respectively. Thus, the DMSO concentration in these experiments did not exceed 0.1% (*v*/*v*).

No visible precipitation of IPA was observed in the culture medium at any working concentration (up to 5 mM) at the beginning or end of the treatment periods (up to 72 h). To assess the selectivity of IPA’s anti-cancer activity, OKF6/TERT-1 cells were exposed to identical IPA concentrations and timepoints (24, 48, and 72 h) as OSCC cell lines in viability and cytotoxicity assays. This parallel treatment design enabled direct comparison of IPA sensitivity between normal and malignant cells.

### 4.3. MTS Metabolic Activity Assay

Metabolic activity was determined using the CellTiter 96^®^ AQueous One Solution Cell Proliferation Assay (MTS assay; Cat. No. G3580, Promega, Madison, WI, USA) according to the manufacturer’s instructions. HSC-3 and SCC-25 cells were plated in 96-well culture plates at 8 × 10^3^ cells per well and incubated for 24 h to allow cell attachment. Cells were then exposed to 0–5 mM IPA for 24, 48, and 72 h. Subsequently, 20 µL MTS reagent was added to each well containing 100 µL culture medium, and the plates were further incubated for 2 h at 37 °C. OKF6/TERT-1 cells were seeded and treated under identical conditions to enable direct comparison of viability responses. All three cell types (OKF6/TERT-1, HSC-3, SCC-25) were processed in parallel using the same assay protocols, reagents, and timepoints. The absorbance was recorded at 490 nm using a microplate spectrophotometer. Background-corrected absorbance values were normalized to the untreated control, and metabolic activity was expressed as a percentage of control. IC50 values were derived from dose–response curves using nonlinear regression analysis with a four-parameter logistic model (log[inhibitor] vs. normalized response—variable slope) generated in GraphPad Prism 8.0. All experiments were repeated independently three times.

### 4.4. Lactate Dehydrogenase Release Assay

To estimate membrane injury and treatment-associated cytotoxicity, culture supernatants were collected after IPA exposure and analyzed using a colorimetric lactate dehydrogenase (LDH) assay kit (E-BC-K1302-M, Elabscience, Houston, TX, USA) according to the manufacturer’s protocol. Absorbance was read spectrophotometrically, and relative lactate dehydrogenase release was normalized to vehicle-treated controls.

HSC-3 and SCC-25 cells were seeded in 96-well plates at a density of 8 × 10^3^ cells/well in 100 µL of complete medium and allowed to attach for 24 h at 37 °C in a humidified incubator containing 5% CO2. Following attachment, the cells were treated with increasing concentrations of IPA (0, 0.25, 0.5, 1, 2.5, and 5 mM) for 24 h and 48 h. At the end of each treatment period, the culture plates were centrifuged at 300× *g* for 5 min to sediment detached cells and cellular debris, and 50 µL of supernatant from each well was carefully transferred to a new flat-bottom 96-well plate. For the preparation of the maximum LDH release control, separate wells were incubated with the kit-supplied lysis solution for the final 30 min before supernatant collection, whereas untreated cells served as the low-control group.

After sample transfer, 50 µL of freshly prepared LDH reaction mixture was added to each well containing culture supernatant, mixed gently, and incubated for 30 min at room temperature protected from light. The enzymatic reaction was stopped by adding the stop solution supplied with the kit, and absorbance was measured at 450 nm using a microplate reader, with 650 nm used as the reference wavelength when applicable. Background absorbance from cell-free medium was subtracted from all readings. Cytotoxicity was calculated as the percentage of LDH release relative to the maximum-release control using the following formula:

(1) Cytotoxicity (%) = [(experimental LDH release − low control)/(maximum LDH release − low control)] × 100

In parallel, LDH release values were also normalized to the untreated control to express fold-change in membrane damage. Each condition was analyzed in at least six technical replicates, and all experiments were repeated independently three times.

### 4.5. Caspase-3 Activity Assay

To determine whether the cytotoxic effects of IPA were associated with activation of the caspase-3-associated response, caspase-3 activity was quantified using a colorimetric caspase-3 assay kit (Cat. No. ab39401, Abcam, Cambridge, UK) according to the manufacturer’s instructions. HSC-3 and SCC-25 cells were seeded in 6-well plates at a density of 2.5 × 10^5^ cells/well and allowed to attach overnight under standard culture conditions (37 °C, 5% CO_2_). After 24 h, the cells were exposed to IPA at the indicated concentrations (0, 0.5, 1, 2.5, and 5 mM) for 24 h. At the end of treatment, both floating and adherent cells were collected, washed twice with ice-cold phosphate-buffered saline (PBS), and lysed in the chilled lysis buffer provided with the kit. Cell lysates were incubated on ice for 10–15 min and then centrifuged at 10,000× *g* for 10 min at 4 °C to remove insoluble material. The resulting supernatants were transferred to fresh tubes, and the total protein concentration of each sample was determined using a BCA protein assay to ensure equal protein input for the enzymatic reaction.

For the caspase-3 activity assay, an equal amount of total protein (100 µg per sample) was added to a 96-well microplate and incubated with the caspase-3-specific colorimetric substrate Ac-DEVD-pNA (Acetyl-Asp-Glu-Val-Asp p-nitroanilide) in reaction buffer containing dithiothreitol (DTT) in a final reaction volume of 100 µL. Ac-DEVD-pNA is a synthetic caspase-3-specific chromogenic substrate that is cleaved to release p-nitroaniline (pNA), enabling quantification of caspase-3 activity. The reaction mixtures were incubated for 1–2 h at 37 °C, protected from direct light, to allow cleavage of the substrate by active caspase-3. Liberation of p-nitroaniline (pNA) was quantified by measuring absorbance at 405 nm using a microplate reader. Blank wells containing assay buffer and substrate without protein lysate were included for background correction. Caspase-3 activity was expressed as fold change relative to the untreated control group after normalization of background-subtracted absorbance values. Each condition was analyzed in triplicate wells within each experiment, and three independent experiments were performed.

### 4.6. Wound-Healing Migration Assay

Cell migratory capacity was evaluated using a wound-healing migration assay. HSC-3 and SCC-25 cells were seeded into 6-well plates at a density of 4 × 10^5^ cells/well and cultured in complete medium until they reached approximately 90–95% confluence. After monolayer formation, a uniform linear scratch was generated in the center of each well using a sterile 200 µL pipette tip. Detached cells and debris were gently removed by washing the wells twice with PBS. The culture medium was then replaced with serum-reduced medium (1% fetal bovine serum) containing the established fixed concentrations of IPA (300 µM for HSC-3 and 400 µM for SCC-25) or vehicle control. These sub-lethal concentrations were utilized to ensure that any observed migration defects were not primarily due to acute cytotoxicity.

Immediately after scratching (0 h), images of the wound area were captured using an inverted phase-contrast microscope at predefined positions. The same fields were photographed again after 24 h and 48 h of treatment. To ensure consistent image acquisition, the underside of each plate was marked to identify identical regions for serial imaging. Image analysis and wound width measurements were performed in a blinded manner, using ImageJ software (Version 1.54p), to ensure objective evaluation of migration capacity. The wound area was quantified by measuring the cell-free gap at each timepoint, and migration was expressed as the percentage of wound closure relative to the initial wound width according to the following formula:

(2) Wound closure (%) = [(wound area at 0 h − wound area at indicated timepoint)/wound area at 0 h] × 100

For each well, measurements were obtained from at least three independent fields, and the mean value was used for statistical analysis. Each condition was tested in triplicate wells, and three independent experiments were performed.

### 4.7. Biochemical Assays for Metabolic Adaptation

Glucose consumption and lactate secretion were quantified in conditioned medium using colorimetric assay kits (Abcam, Cat. No. ab65333, Abcam, Cat. No. ab65331, Abcam, Cat. No. ab83355). Intracellular adenosine triphosphate content was assessed using a commercial kit according to the manufacturer’s instructions. Conditioned-medium pH was measured as a bulk-medium parameter under the experimental culture conditions. Because these measurements were not normalized to DNA or total protein measured at harvest, they were interpreted as bulk biochemical readouts rather than definitive per-cell measurements of glycolytic flux or extracellular proton flux.

### 4.8. Measurement of Intracellular Reactive Oxygen Species by Spectrophotometric Method

Intracellular ROS levels were quantified using a spectrophotometric/colorimetric ROS detection approach based on oxidation of the cell-permeable probe supplied in the assay kit (Abcam, Cat. No. ab113851), according to the manufacturer’s instructions. HSC-3 and SCC-25 cells were seeded in 96-well plates at a density of 1 × 10^4^ cells/well in 100 µL of complete culture medium and allowed to attach for 24 h under standard culture conditions (37 °C in a humidified incubator with 5% CO_2_). After attachment, cells were treated with the indicated concentrations of IPA (0, 0.5, 1, 2.5, and 5 mM) for 24 h. At the end of the incubation period, the culture medium was carefully aspirated, and the cells were gently washed twice with warm PBS to remove residual medium and serum components that could interfere with oxidation-dependent signal generation.

For ROS determination, cells were incubated with 100 µL of the working ROS detection reagent prepared in serum-free medium and protected from direct light for 30 min at 37 °C. During this period, the probe diffused into the cells and was converted into a detectable oxidized product in proportion to intracellular oxidant burden. After incubation, excess reagent was removed, and cells were washed three times with PBS to minimize nonspecific background signal. Fresh assay buffer was then added to each well, and absorbance was measured immediately using a microplate reader at the wavelength recommended by the assay manufacturer (typically 490–520 nm, depending on the kit format). Blank wells containing assay reagent but no cells were included on each plate for background subtraction, whereas untreated cells served as the baseline control group.

To confirm assay responsiveness, an independent set of wells was exposed to hydrogen peroxide (H_2_O_2_, 100 µM for 30 min) as a positive control for oxidative stress before ROS measurement. The signal obtained from H_2_O_2_-treated wells was used to verify adequate analytical sensitivity of the assay system. Raw absorbance values were corrected by subtracting the mean blank value from each reading. Because treatment-related changes in viable cell number may influence total signal intensity, ROS values were normalized to metabolic activity data obtained from parallel MTS assay plates treated under identical experimental conditions. Relative intracellular ROS levels were calculated using the following formula:

(3) Relative ROS = [(Absorbance treatment − Absorbance blank)/Viability treatment] ÷ [(Absorbance control − Absorbance blank)/Viability control]

The normalized ROS level of the untreated control group was assigned a value of 1.0, and all other groups were expressed as fold change relative to control. Each treatment condition was analyzed in at least three replicate wells per experiment, and all experiments were repeated independently three times.

### 4.9. Quantitative Real-Time Polymerase Chain Reaction

Total RNA was extracted from all experimental groups using the Total RNA Purification Kit (Hopegen Biotech, Hangzhou, China; Cat. No. TR01) in accordance with the manufacturer’s protocol. RNA yield and purity were assessed with a NanoDrop spectrophotometer (Thermo Fisher Scientific, Waltham, MA, USA), and samples with an A260/A280 ratio of approximately 2.0 were considered sufficiently pure for further analysis.

For complementary DNA (cDNA) synthesis, 1 μg of total RNA was reverse-transcribed using the OneScript™ Plus cDNA Synthesis Kit (Abcam, Cambridge, UK; Cat. No. G236). Reverse transcription was carried out in a thermal cycler under the following conditions: 15 min at 50–55 °C, followed by 5 min at 85 °C.

Quantitative real-time PCR (qRT-PCR) was conducted using 2× SYBR Green Real-Time PCR Master Mix (Nepenthe, Kocaeli, Türkiye, NP0401021100) on a LightCycler 480 Real-Time PCR System (Roche, Basel, Switzerland). GAPDH was used as the sole endogenous reference gene for exploratory relative-expression analysis. Formal reference-gene stability analysis using multiple housekeeping genes was not performed. Relative gene expression levels of HIF1A, GLUT1, HK2, LDHA, MCT4, MYC, CCND1, CDH1, SOD2, and CAT were calculated using the 2^(−ΔΔCt)^ method, with GAPDH serving as the normalization reference. All reactions were performed in technical triplicate, and each experiment was repeated independently three times. Primer sequences are provided in Table 1.

### 4.10. Conceptual Pathway Modeling

The conceptual pathway model (Figure 5D and Figure 6) was constructed by integrating the observed transcriptional changes (qRT-PCR data) and functional assay results (metabolic assays, ROS generation, and migration) into established biological frameworks of glycolysis, oxidative stress, and epithelial–mesenchymal transition. It represents a synthesized interpretation of the experimental findings rather than a direct bioinformatic output.

### 4.11. Statistical Analysis

All experiments were performed in three independent biological replicates, with at least three technical replicates per condition in each independent experiment. The data are presented as the mean ± standard deviation (SD). Statistical analysis was performed using GraphPad Prism 8 software (GraphPad Software, San Diego, CA, USA). Differences between groups were analyzed using a two-tailed Student’s t-test or one-way analysis of variance (ANOVA) followed by Tukey’s post hoc test. A *p*-value < 0.05 was considered statistically significant.

## 5. Conclusions

In summary, this study demonstrates that the microbiota-derived metabolite IPA exerts multi-level suppression of OSCC cell fitness through the coordinated disruption of metabolic plasticity and modulation of epithelial–mesenchymal transition markers. IPA reduced metabolic activity, glucose consumption, lactate secretion, and intracellular ATP while simultaneously increasing oxidative stress markers, caspase-3 activity, and CDH1 expression. Importantly, these effects were selective for malignant cells, as OKF6/TERT-1 cells exposed to identical IPA concentrations maintained near-complete viability. These findings indicate that IPA targets the adaptive mechanisms—glycolytic reprogramming and EMT—that enable OSCC progression. The more pronounced response observed in HSC-3 cells suggests that metabolically aggressive phenotypes are particularly vulnerable to IPA-mediated stress. While this in vitro study cannot address stromal interactions, immune modulation, or in vivo pharmacokinetics, it provides a mechanistic foundation for investigating microbiota-derived metabolites as therapeutic agents in oral cancer. Future studies should validate these findings in three-dimensional organoid models and xenograft systems and explore potential synergistic effects with conventional therapies. This microbiota-informed metabolic framework may open novel therapeutic avenues for OSCC management and underscores the importance of host-microbe interactions in oral carcinogenesis.

## Figures and Tables

**Table 1:** Primer sequences used for qRT-PCR.

Gene	Primer Direction	Sequence (5′–3′)	Product Length (bp)	Tm (°C)
HIF1A	Forward Reverse	GATCAGCCTGGATCTTCAGGG TCAGTGGTGGCTGTATGATGCT	132	60.1 60.0
GLUT1	Forward Reverse	GTATTGCCCTGGAAGGGAAGC GTATTGCCCTGGAAGGGAAGC	118	59.8 60.2
HK2	Forward Reverse	GAGCCACCACTCACCCTACT GCCACTGTTCTCTCGTTCAGT	145	60.0 59.9
LDHA	Forward Reverse	GGACACAGACTCTCCATCGG TCCTCGTGATACATGGTCTCG	128	60.1 59.7
MCT4	Forward Reverse	GTGGCTGTTCATTATGCCATCG CCTTCTCGCTCTCGTTGATACTC	136	59.9 60.0
MYC	Forward Reverse	CCTGGTGCTCCATGAGGAGAC CAGACTCTGACCTTTTGCCAGG	151	60.2 60.1
CCND1	Forward Reverse	TCTACACCGACAACTCCATCCG TCTGGCATTTTGGAGAGGAAGTG	120	59.8 60.0
CDH1	Forward Reverse	GCCTCCTGAAAAGAGAGTGGAAG CTGGTGCTAAAGTGATGTCCGT	112	59.7 59.9
SOD2	Forward Reverse	TTACGACAACGCTGCCGATACT GGAACTCTCAATTCCTCACCCACT	125	60.0 59.8
CAT	Forward Reverse	GTGCGGAGATTCAACACTGCCA CGGCAATGTTCTCACACAGACG	108	60.1 60.0
GAPDH	Forward Reverse	GGCCAAGATCATCCATGACAACT ACCAGGACATGAGCTTGACAAAGT	100	55.27 55.68

## Data Availability

The original contributions presented in this study are included in the article/Supplementary Material. Further inquiries can be directed to the corresponding author.

## Supplementary Materials

The following supporting information can be downloaded at: https://www.mdpi.com/article/10.3390/ijms27198867/s1.
