# Supplementary material for: Indole-3-Propionic Acid Disrupts Glycolytic Adaptation and Promotes Oxidative Stress-Associated Injury in Oral Squamous Cell Carcinoma
Source: Int J Mol Sci. 2026 Oct 5;27(19):8867. doi: 10.3390/ijms27198867 (PMC13664853; doi:10.3390/ijms27198867)
Supplement: Supplementary file 1 [file ijms-27-08867-s001.zip › ijms-4545843-supplementary.pdf]

Supplementary

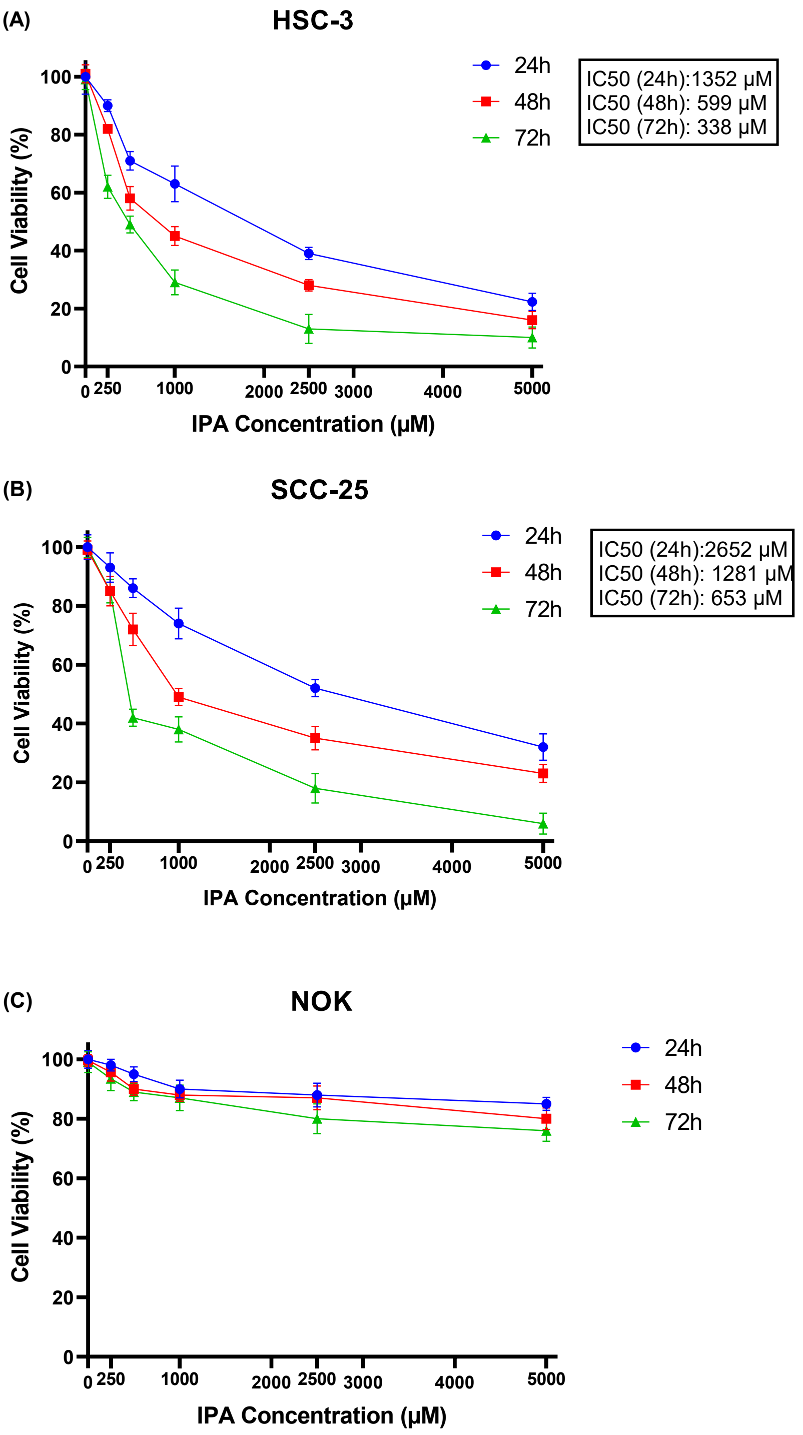

Supplementary Figure S1. Dose-response curves of IPA on cell viability determined by MTS assay. (A) HSC-3, (B) SCC-25, and (C) NOK cells were treated with increasing concentrations of IPA (0–5000 μM) for 24 h (blue), 48 h (red), and 72 h (green), and viability was assessed using the MTS assay. Data are presented as mean ± SD (n = 3 independent biological replicates). IC50 values were calculated using nonlinear regression analysis with a four-parameter logistic model (log[inhibitor] vs. normalized response — variable slope). HSC-3 IC50 values: 1352 μM (24 h), 599 μM (48 h), 338 μM (72 h). SCC-25 IC50 values: 2652 μM (24 h), 1281 μM (48 h), 653 μM (72 h). NOK cells showed no significant viability loss (IC50 > 5000 μM) at any timepoint, confirming the cancer-selective cytotoxicity of IPA.
